# Quality of the Antibiotics—Amoxicillin and Co-Trimoxazole from Ghana, Nigeria, and the United Kingdom

**DOI:** 10.4269/ajtmh.14-0539

**Published:** 2015-06-03

**Authors:** Ifeyinwa Fadeyi, Mirza Lalani, Naiela Mailk, Albert Van Wyk, Harparkash Kaur

**Affiliations:** London School of Hygiene & Tropical Medicine, London, United Kingdom

## Abstract

Little is known about the quality of antibiotics despite being in high demand globally. Thirty five samples (27 brands) of the antibiotics amoxicillin (*N* = 20; 16 brands) and co-trimoxazole (*N* = 15; 11 brands), manufactured in six countries (China, Ghana, India, Ireland, Nigeria, and United Kingdom), were purchased in Ghana, Nigeria, and the United Kingdom. Their quality was assessed using German Pharma Health Fund (GPHF) MiniLab^®^ as the screening tool—two capsules of amoxicillin (10%) and two tablets of co-trimoxazole (20%) failed the thin-layer chromatography (TLC) test. Definitive drug quality was measured using high-performance liquid chromatography–photodiode array detection (HPLC-PDA) for content of the stated active pharmaceutical ingredients (APIs) and bioavailability was determined with in vitro dissolution testing. All the samples of amoxicillin complied with U.S. Pharmacopeia (USP) tolerance limits, but 60% tablets of co-trimoxazole (purchased in Ghana and Nigeria) did not. There was disparity in the results obtained for co-trimoxazole and amoxicillin samples using the MiniLab^®^ TLC tests. This highlights the need to invest in techniques such as HPLC-PDA and dissolution testing alongside the screening tests for assessing drug quality.

## Introduction

Antibiotics such as co-trimoxazole (sulfamethoxazole/trimethoprim—a dihydrofolate reductase inhibitor and a sulphonamide) and amoxicillin (a β-lactam) are used globally in the public health sector for treatment of bacterial infections. Antibiotics in general are crucial for use as chemotherapeutic agents in treating bacterial infections and microbe-borne diseases making them vital for the prevention of mortality in pneumonia, diarrheal diseases, human immunodeficiency virus (HIV), tuberculosis, and malaria.^1^–^3^ Co-trimoxazole is commonly used as prophylaxis against secondary bacterial infections among HIV-1-infected tuberculosis patients for whom it has shown a marked reduction in morbidity and mortality rates.^4^ Amoxicillin is a broad-spectrum antibiotic used for the treatment of several bacterial infections.^5^

There is widespread concern about the quality of antimalarial drugs, with up to 35% poor quality antimalarials reported to be in malaria-endemic countries.^6^,^7^ However, little is known about the quality of antibiotics, despite being in high demand globally. Manufacturers of “counterfeit”^8^ drugs target economically profitable medicines, as well as those that have high volume sales. Over a period of 5 years, 2006–2010, 1.34 billion antibiotic prescriptions were dispensed in the United States.^9^ Data are limited from the developing world where first-line antibiotics can be easily obtained without prescription from pharmacies, grocery shops, and even mobile drug peddlers.^10^,^11^

The use of poor quality drugs can lead to poor treatment outcomes, waste of financial resources by prolonging illnesses, increase the potential of recrudescence, and propagate the development of drug resistance.^12^ Instances such as these reduce consumer confidence in health systems, health professionals, and the pharmaceutical industry.

A limited number of scientific investigations have assessed the prevalence of poor quality antibiotics.^13^,^14^ A study carried out of antibiotics purchased in the middle east and north Africa found that there was a substantial amount of locally produced amoxicillin that did not comply with drug monographs in the U.S. Pharmacopeia (USP).^15^ Indeed data available on the quality of anti-infective drugs mainly focus on antimalarial drugs.^16^

The assessment of the quality of drugs in developing countries that do not have a medicines quality control laboratory (MQCL) is often a two-stage process. The first involves the screening of drugs, which can be carried out in the country where the drugs have been purchased provided they have a portable laboratory, in particular the German Pharma Health Fund (GPHF) MiniLab^®^.^17^ The MiniLab^®^ contains four basic tests: visual inspection, tablet/capsule disintegration, colorimetric tests,† †According to the manufacturer of the MiniLab the colorimetric test is no longer recommended as part of the screening process. and thin-layer chromatography (TLC). The MiniLab^®^ is regarded as a simple and inexpensive testing kit requiring minimal training and no electricity to operate. The U.S. Agency for International Development (USAID) through its implementing partner USP identifies the MiniLab^®^ as a key aspect of its Promoting the Quality of Medicines (PQM) program in several developing countries.^18^

The second stage is to detect the ingredients in each drug sample at a MQCL using the technique of high-performance liquid chromatography (HPLC) to then quantify the amount of active pharmaceutical ingredients (APIs). HPLC is regarded as the gold standard for drug quality analysis as it offers accuracy, specificity, and precision in quantifying the amount of stated API detected or its absence. In vitro, dissolution testing offers valuable prediction of the in vivo bioavailability and bioequivalence of tablets and capsules. Dissolution tests measure the amount of drug released into the dissolution media with time following detailed protocols (official monographs) set out for most drugs in pharmacopeias (e.g., European, British, USP, World Health Organization [WHO] International). The protocols outline the details of the test conditions (dissolution buffer/solvents, stirring speed, tolerance levels of the API, and temperature for the assay). Even if the quantity of API in a medicine is within pharmacopeia's tolerance limits for content, the amounts released (bioavailability) may be lower giving poor dissolution characteristics. The dissolution tests require sophisticated apparatus as well as the analytical equipment such as HPLC, and most crucially the latter analysis requires the reference standards of the compounds being tested for calibrating the equipment, which can be both expensive and difficult to obtain.^19^

This study was undertaken to determine the quality of antibiotics among varying brands of amoxicillin and co-trimoxazole purchased in Kintampo town (Ghana); Agbor town, Delta state, and Lagos city (Nigeria); and Milton Keynes (United Kingdom). Drug quality was assessed at our laboratory in London using the GPHF MiniLab^®^ as the screening tool, HPLC–photodiode array detection (PDA) to measure content of formulations and the in vitro dissolution testing adhering to USP monographs to decide on the bioavailability of the antibiotic samples.

## Materials and Methods

### Sampling the antibiotics.

**Figure 1.**
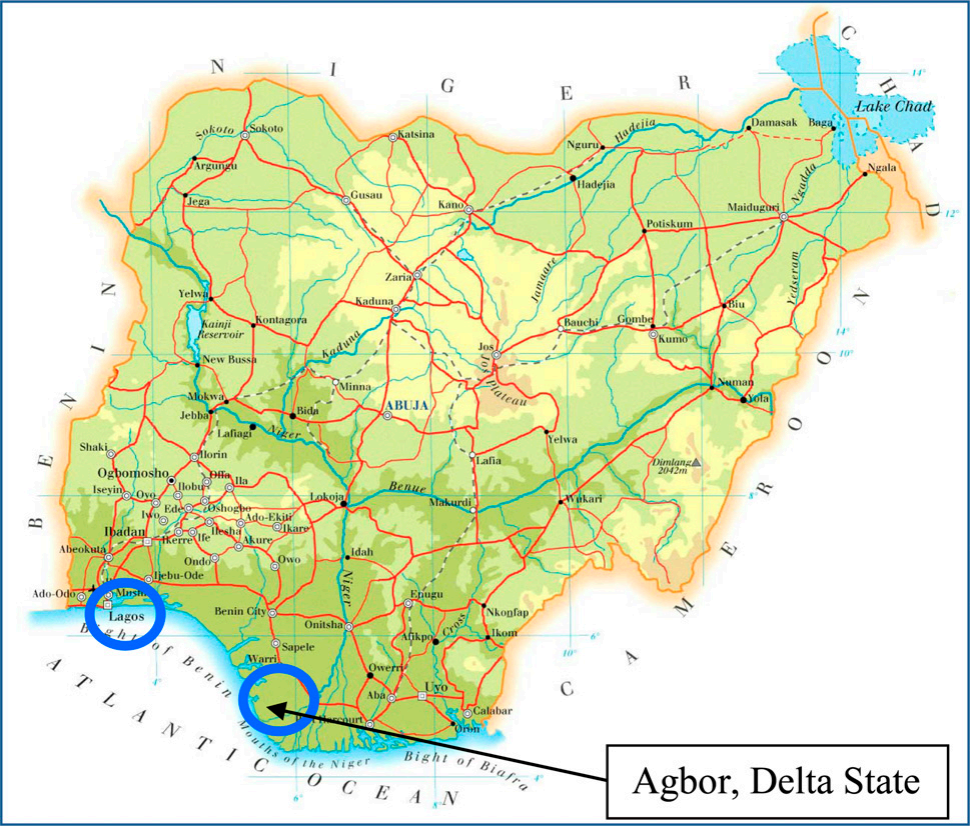
Map of Nigeria.^20^

Samples of amoxicillin and co-trimoxazole were purchased from a variety of drug outlets including; pharmacies, licensed chemical stores, and drug vendors in Ghana, Nigeria, and United Kingdom using a convenience sampling method with an overt approach. This sampling method involves the purchaser buying the medicines without specific guidance on which outlets to visit. All outlet owners in each country were informed of the nature of the study prior to purchasing the samples. The places in Nigeria where samples were purchased are shown on the map in Figure 1

**Figure 2.**
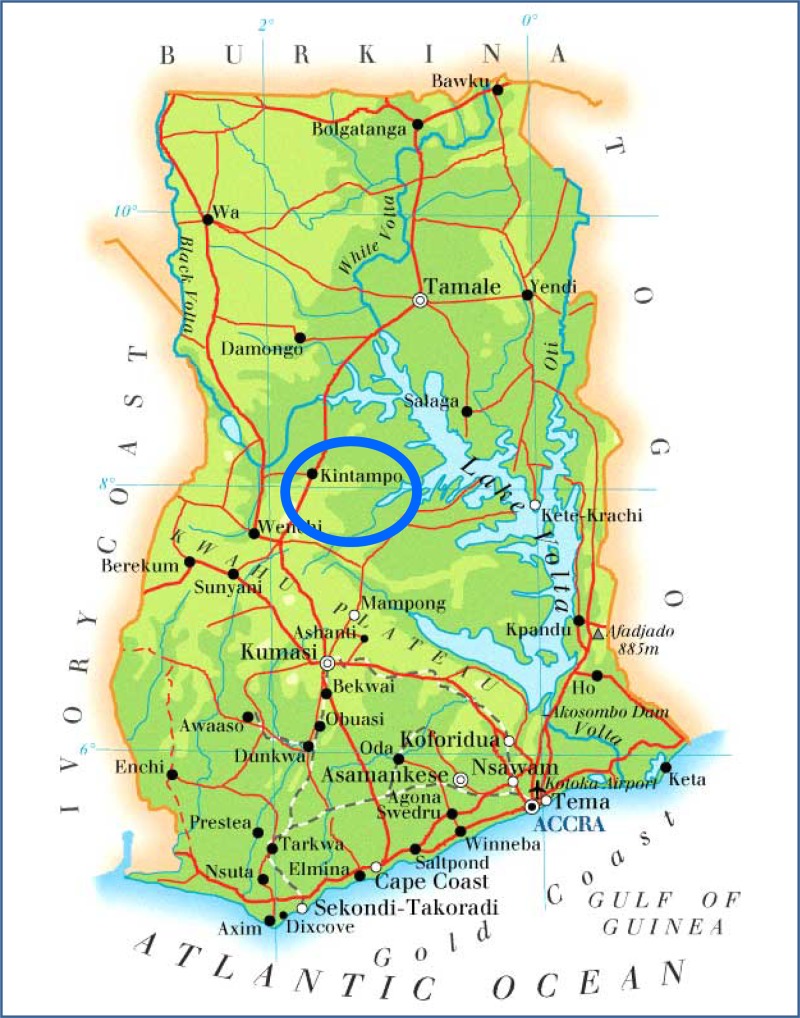
Map of Ghana.^21^

.^20^ In Ghana, samples were purchased in Kintampo, a major town in the central part of the country (Figure 2

).^21^ Samples from Milton Keynes (United Kingdom) were donated by a local pharmacy. Samples of the same brand, but with different batch numbers were classified as an individual sample. A total of 20 samples (16 brands) of amoxicillin and 15 samples of co-trimoxazole (11 brands) were acquired by asking the outlet for all the brands of available antibiotics that they stocked at the time of sampling.

### Ethics approval.

The London School of Hygiene and Tropical Medicine research ethics committee approval (Ref: 5804) was secured for the purchase of the antibiotics.

### Sample logging.

The packaging and/or blister packs of all the samples were logged in the data collection tool (Epi Info version 3.5),^22^ which is a public domain statistical software for epidemiology developed by Centers for Disease Control and Prevention in Atlanta, GA, to capture information including brand name, stated APIs, dose form, country of sample collection, date of purchase, name of stated manufacturer, country of manufacture, batch number, date of manufacture, expiry date, and number of capsules/tablets per packet (pack size). Each sample was placed in an individual ziplock bag and given a bar code. All samples were logged onto a database, and tablets weighed and measured prior to laboratory analysis. Drug quality analyses were conducted on a minimum of two tablets or two capsules.

### Drug quality analysis.

All samples were assessed for their quality in our laboratory in London using the GPHF MiniLab^®^ as the screening tool.^17^ The amount of stated API for the content analysis and release over time for the in vitro dissolution testing was measured using HPLC-PDA, following USP monographs.^23^

#### Visual inspection.

All the packaging and blister packs of formulations were digitally scanned to record any abnormal spelling or unduly faded color of the packaging as a result of being stored in direct sunlight and/or high humidity. Each formulation (tablet or capsule) was also weighed and measured using electronic digital calipers.

#### Drug quality screening test (GPHF MiniLab^®^).

All the samples were tested using two of the tests in the MiniLab^®^ following the manual instruction for each drug.^17^

- Colorimetric test detects the presence of the stated API.
- TLC test identifies the APIs present in each sample.

#### HPLC-PDA conditions.

Reference standards of amoxicillin, sulfamethoxazole, and trimethoprim were purchased from Sigma-Aldrich, Dorset, United Kingdom. All equipment and solvents used were purchased from Thermofisher Scientific, Hemel Hempstead, UK.

**Figure 3.**
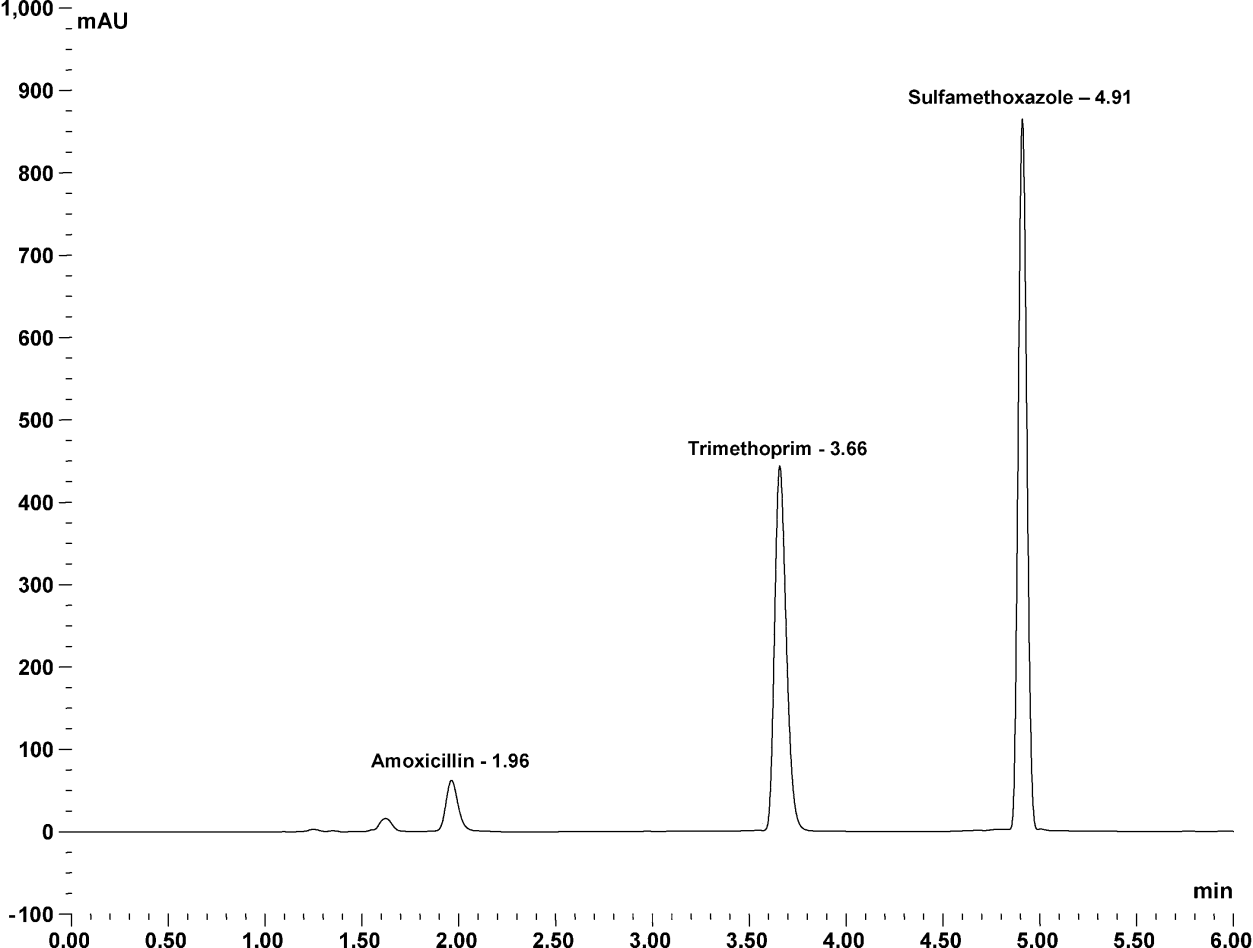
High-performance liquid chromatography (HPLC) chromatogram of amoxicillin and co-trimoxazole.

HPLC analyses were carried out using a Dionex Ultimate 3000 HPLC system. In brief, samples were separated on an Acclaim 120, C_18_, 5-μm analytical column (4.6 × 150 mm) from Fisher Scientific, Leicestershire, United Kingdom, with gradient elution from 100% solvent A (20 mM ammonium formate [pH 2.7]) to 100% solvent B (acetonitrile) over 6 minutes at a flow rate of 1.4 mL/min. The photo-diode array detector (UV-PDA; DAD 3000) was set at 275 nm (better resolution of the peak of amoxicillin is achieved at 230 nm). Figure 3 shows the separation of the three compounds. Peak identity of amoxicillin, sulfamethoxazole, and trimethoprim was confirmed by measuring the retention time, spiking the sample with commercially available standards and determination of absorbance spectra using the UV-PDA. Calibration curves of each compound were generated by Thermofisher Scientific Dionex Chromeleon 7.2 chromatography data system software (Thermofisher Scientific, Hemel Hempstead, UK) using known amounts of the relevant standard. The coefficient of determination for amoxicillin was 99.39%, for trimethoprim 97.83%, and for sulfamethoxazole 99.60%. Results for the content analysis are expressed as a percentage of the detected API in a given sample from the stated dose on the packaging. For dissolution testing, the amount of API released over time was monitored using our in-house HPLC-PDA method for the analysis of the aliquot, to determine compliance with USP tolerance limits.

#### Content analyses.

Content of the formulations of amoxicillin and co-trimoxazole was measured by dissolving each formulation in solvent (0.1 M HCl for amoxicillin and methanol for co-trimoxazole) to give a 1 mg/mL solution that was further diluted to obtain a 0.5 mg/mL solution, and analyzed using our in-house HPLC method outlined above.

The USP rules for content analysis stipulate that for each tablet of amoxicillin 90–120% stated API should be measured, and for co-trimoxazole it should be 93–107%.

#### Dissolution analyses.

The quality of the formulations of amoxicillin and co-trimoxazole was determined using the Pharma Test PT 017 dissolution apparatus (Pharma Test GroupPharma, Hainburg, Germany) following the in vitro dissolution testing protocols detailed in the monographs outlined in the USP (USP 24)^23^ and measuring the API released using our HPLC method outlined above. The USP dissolution tolerance rules stipulate that not less than 80% amoxicillin should dissolve in the media (water) in 60 minutes (i.e., 0.22 mg/mL for a 250 mg dose and 0.45 mg/mL for a 500 mg dose), and not less than 70% of each component of sulfamethoxazole /trimethoprim should dissolve in the media (0.1 N HCl) in 60 minutes (i.e., 0.06 mg/mL for a 400/80 mg dose, and 0.12 mg/mL for a 800/160 mg dose).

### Sensitivity and specificity calculations.

Although the sample size in this pilot study is small, we have nevertheless analyzed 27 brands of antibiotics manufactured in 6 countries (China, Ghana, India, Ireland, Nigeria, and United Kingdom) and purchased in three countries using MiniLab^®^ as a screening tool and HPLC as the gold standard test. The MiniLab^®^ tests results were compared with the HPLC content analysis results to assess the sensitivity and specificity of the tests to detect non-adherent samples. 95% confidence intervals were calculated using exact binomial distribution.

## Results

The sample information and analyses results for each sample of antibiotic are shown in Tables 1 and 2.

### Visual inspection.

Majority of the brands of antibiotics analyzed in this study were labeled as having been produced as generics in China, Ghana, India, Ireland, and Nigeria. We were not successful in obtaining the original packets of generics from manufacturers to help us to compare and distinguish any differences.

### GPHF MiniLab^®^ drug quality screening.

Results of the MiniLab^®^ tests are classified as pass in the colorimetric test if the drugs produce the expected color. Similarly for a drug to be classified as pass using the TLC test, the spot of the sample should migrate at the same rate as that for the “working standard solution 80%” of the API, which represents the lowest acceptable limit.^17^ All 20 samples of amoxicillin produced the dark red-brown solution as per the MiniLab^®^ manual for each of these drugs and were classified as pass whereas two samples, purchased in Ghana, failed the TLC test (Table 1). A further set of tablets (two per packet) were tested from the failed samples to make sure that this was not a technician error and the samples again failed in the test. All 15 samples of co-trimoxazole produced a brown solution hence passing the colorimetric test, and two samples, one from Ghana and one from Nigeria, failed the TLC test (Table 2).

#### HPLC content analysis and dissolution testing.

#### Content analysis.

All the capsules of amoxicillin, except one purchased in Nigeria, were compliant in the content analysis test. One co-trimoxazole tablet (obtained in the United Kingdom) was compliant with the content analysis test, while 14 samples purchased in Ghana and Nigeria were not. The latter contained between 63% and 90% sulfamethoxazole and 38% and 88% trimethoprim.

#### Dissolution testing.

All samples of amoxicillin released the expected amount of API within time and met the USP tolerance limits. Of the 15 co-trimoxazole purchased, 6 out of 15 (40.0%) samples (two from Ghana and four from Nigeria) met USP tolerance limits but 9 out of 15 (60%; three from Ghana and six from Nigeria) did not.

### Sensitivity and specificity calculations.

Performance of MiniLab^®^ tests to detect samples noncompliant with HPLC content analysis are reported in Table 3. Although numbers are very small, none of the MiniLab^®^ tests indicate that these tests will not be successful at detecting samples that failed the HPLC content analysis, for either amoxicillin or co-trimoxazole. However, the MiniLab^®^ does demonstrate a low sensitivity (14%) for co-trimoxazole.

## Discussion

Sulfonamides and β-lactam antibiotics have saved countless lives since their discovery in the 1930s. However, there has since been a disengagement of most pharmaceutical companies from antibiotic research as a result of economic and regulatory challenges. The number of multinational pharmaceutical companies actively engaged in antibiotic research has fallen from 18 in 1990 to just 4 in 2011—AstraZeneca, Novartis, GlaxoSmithKline (GSK), and Sanofi-Aventis,^24^ with countless numbers of generic antibiotics being manufactured globally.

Unremitting treatment using antibiotics is known to select bacterial strains with physiologically or genetically enhanced abilities to withstand high concentrations of antibiotics.^26^ Misuse of antibiotics through unnecessary overprescribing and suboptimal dosing engenders the development of resistance. Of greater alarm for public health globally is the emergence of “superbugs” that are extremely resistant to the majority of antibiotics.^27^ The threat of superbugs added to the disengagement of pharmaceutical companies makes the maintenance of good quality antibiotics of paramount importance.^26^,^28^ Drug quality monitoring requires effective tools and regulatory systems to ensure all available drugs reaching the patients are quality assured. A systematic literature review found there to be a prevalence of poor quality antimicrobial medicines, with the majority of studies focusing on antimalarial drugs in sub-Saharan Africa and Asia reporting that up to 30% purchased predominately using convenience sampling in 21 sub-Saharan countries, failed the chemical content analysis.^29^

The GPHF MiniLab^®^ colorimetric test produced the expected change in color for all the samples of antibiotics tested. Although, the TLC test will pass all samples that contain greater than 80% API, it did not result in the expected migration of spots for two samples of amoxicillin bought in Ghana (one was stated to be manufactured in United Kingdom and the other in Ghana) even after retesting using two other tablets in the packet. Furthermore, two samples of co-trimoxazole bought and stated to be manufactured in Ghana and Nigeria, respectively, also failed the TLC test.

Co-trimoxazole is the recommended drug by the WHO for use as a prophylactic therapy to decrease the burden of bacterial infections for people living with HIV and tuberculosis.^30^ Indeed, in Nigeria it is indicated for the prevention of several secondary bacterial and parasitic infections in HIV-infected individuals.^31^ It is therefore worrying that in our study, 9 out of the 15 tablets of co-trimoxazole subjected to dissolution testing did not meet the USP tolerance limits. These subtherapeutic tablets were all bought in Ghana and Nigeria, stating that they were manufactured in those countries, except for one that states it was manufactured in India. These tablets did not contain an acceptable amount of the stated API (93–107%) when tested for content with amounts of sulfamethaxozale ranging between 63% and 90% and trimethoprim ranging between 38% and 88%.

The quality of amoxicillin assessed using both the MiniLab^®^ and the HPLC analysis for content produced some anomalies. Two samples from Ghana failed the MiniLab^®^ TLC test but were found to be compliant by HPLC content analysis. One sample from Nigeria passed the MiniLab^®^ TLC test but was found to be noncompliant by HPLC content analysis. The MiniLab^®^ was more useful for testing amoxicillin but disparity was found between the results obtained when assessing the quality of co-trimoxazole using the MiniLab^®^ TLC test and the HPLC content analysis. The TLC method detected only two noncompliant samples out of the total 15, whereas HPLC content analysis determined 14 samples of co-trimoxazole to be noncompliant. This underestimation of poor quality using the TLC test demonstrates the lack of accuracy of the MiniLab^®^ to detect noncompliant samples of certain drugs such as co-trimoxazole in this study. This limitation has been previously reported in a study assessing the quality of antimalarials in sub-Saharan Africa in which the MiniLab^®^ TLC method failed to detect noncompliance (as determined by HPLC) of 59% of 41 samples of artemisinin combination therapy drugs and sulfadoxine–pyrimethamine.^32^ The MiniLab^®^ is a suitable screening tool in the absence of MQCLs; however, it has been reported that it can only detect grossly substandard or counterfeit drugs, which has been highlighted here with co-trimoxazole.^33^ Indeed specificity and sensitivity calculations of our analysis results, even though our sample size was small, support the published findings, as the MiniLab^®^ demonstrates a low sensitivity for co-trimoxazole tablets when compared with content analysis with HPLC. All the samples passed the colorimetric test and only two failed the TLC test. Therefore, the MiniLab^®^ cannot be relied upon unequivocally for drug quality monitoring, and for definitive results precise analytical methods such as HPLC and dissolution testing need to be utilized.^19^ It is worth noting that results provided by the MiniLab^®^ are intrinsically linked to the drug-specific protocol for testing, and so perhaps this is an aspect that needs to be assessed by the manufacturer. Studies with larger sample sizes need to be undertaken with a number of the main classes of anti-infective drugs to determine the sensitivity and specificity of the MiniLab^®^.

Notably no falsified samples were identified in this study, even though the sample size was small, but substandard co-trimoxazole samples containing less than 93% APIs have been detected. These may have been produced following poor manufacturing practices where the facilities have not been certified to have met “Good manufacturing practice” status, which may be the case in resource-poor settings.^34^ The impact of substandard co-trimoxazole at the patient level may be a longer recovery time from infection increasing the burden on health services and the inevitable impact on the productivity of the individuals and/or carer(s). However, at the population level substandard co-trimoxazole may engender drug resistance.

Lack of funds and time entailed that samples were purchased using the convenience method with an overt approach. Convenience sampling is not the method of choice to determine the prevalence of poor quality drugs in a geographical region, and overt sampling may lead to responder bias as the seller is aware of the nature of the study and will provide samples that are more likely to be of good quality.^35^

In conclusion, there has been much attention focused on the quality of antimalarials as well as the quality of antiretroviral drugs for HIV and drugs for the treatment of tuberculosis in parts of southeast Asia and sub-Saharan Africa. However, a concerted effort is needed to also determine the quality of varying brands of antibiotics in various countries. The sheer volume of antibiotics sold daily and their relatively low production cost makes them a vulnerable group of drugs for targeting by counterfeiters, illegitimate internet pharmacies, and those drug manufacturers who use poor manufacturing practices.^32^ The use of screening tools such as the MiniLab^®^ is vital for countries that do not have a MQCL, but our work has shown that it does not work well for one of the two antibiotics that we tested and further investigation is warranted with greater sample number and other antibiotics. This study further endorses the need for developing nations to invest in capacity building integrating national MQCLs. Investment must include improving technical capacity so that drug quality analysis can be conducted using reliable and accurate methods such as HPLC and dissolution testing to be a part of an integrated drug quality surveillance system.

## Figures and Tables

**Table 1:** Sample information and analyses results for amoxicillin

Drug (total no of samples)	Brand; manufacturer; country of manufacture	Country of purchase	Expiry date (month-year)	Strength (mg)	MiniLab^®^ CT	MiniLab^®^ TLC test	HPLC content analysis adherence	Dissolution test compliance
	Co-Trimoxazol; Letap Pharmaceuticals Ltd; Ghana	Ghana	Apr-12	250	Pass	Pass	Yes	Yes
	Amoxicillin; Ayrton Drug Mfg; Ghana	Ghana	Oct-13	250	Pass	Pass	Yes	Yes
	Amoxicillin 250 mg; GR Industries Ltd; Ghana	Ghana	Jan-14	250	Pass	Pass	Yes	Yes
	Amoxylex; Luex Healthcare; United Kingdom	Ghana	Jun-12	250	Pass	Pass	Yes	Yes
	Permoxyl 500; Ernest Chemicals; Ghana	Ghana	Jan-14	500	Pass	Fail	Yes	Yes
	Promox capsules 500 mg; Medreich PLC; United Kingdom	Ghana	Jan-13	500	Pass	Fail	Yes	Yes
	Amoram 500 mg capsules; LPC Medical (UK) Ltd; United Kingdom	Ghana	Oct-12	500	Pass	Pass	Yes	Yes
	Amoxicillin 500 mg; GR Industries; Ghana	Ghana	Apr-12	500	Pass	Pass	Yes	Yes
	Floximox; Evans Medical; Nigeria	Nigeria	Not stated	250	Pass	Pass	No	Yes
Amoxicillin capsules (*N* = 20)	Amoxil; Beecham/Medreich; India	Nigeria	Apr-13	250	Pass	Pass	Yes	Yes
	Reichamox^®^; Medreich; India	Nigeria	Sep-11	250	Pass	Pass	Yes	Yes
	Moxitin-500 Amoxicillin capsules B.P.; Clarion Medical; China	Nigeria	Oct-13	500	Pass	Pass	Yes	Yes
	Amoxicillin 250 mg capsules BP; Milpharm Ltd; United Kingdom	United Kingdom	Aug-12	250	Pass	Pass	Yes	Yes
	Amoxicillin capsules BP; Actavis; United Kingdom	United Kingdom	Nov-11	250	Pass	Pass	yes	Yes
	Amoxil^®^ capsules 250 mg; GSK; United Kingdom	United Kingdom	Jan-14	250	Pass	Pass	Yes	Yes
	Amoxicillin 250 mg capsules; Medreich PLC; England	United Kingdom	Nov-12	250	Pass	Pass	Yes	Yes
	Ranbaxy Amoxicillin; Ranbaxy; Ireland	United Kingdom	Jan-12	250	Pass	Pass	Yes	Yes
	Amoxicillin 500 mg capsules; Athlone Laboratories; Ireland	United Kingdom	Feb-15	500	Pass	Pass	Yes	Yes
	Amoxicillin 500 mg capsules; Accord Healthcare Limited; United Kingdom	United Kingdom	Nov-13	500	Pass	Pass	Yes	Yes
	Amoxicillin capsules; Amoxicillin Trihydrate; Morningside Pharmaceuticals; United Kingdom	United Kingdom	Jul-11	500	Pass	Pass	Yes	Yes
Total passed (%)					20/20 (100.0)	18/20 (90.0)		
Total failed (%)						2/20 (10.0)		
Total compliant with USP tolerance limits (%)							19/20 (95.0)	20/20 (100.0)
Total noncompliant with USP tolerance limits (%)							1/20 (5.0)	

CT = colorimetric test; GSK = GlaxoSmithKline group of companies; HPLC = high-performance liquid chromatography; TLC = thin-layer chromatography; USP = U.S. Pharmacopeia.

The USP content limits state that 90–120% of the stated dose of amoxicillin must be measured for the tablets to be classified as compliant.

**Table 2:** Sample information and analyses results for co-trimoxazole

Drug (total no of samples)	Brand; manufacturer; country of manufacture	Country of purchase	Expiry date (month-year)	Strength (mg)	MiniLab^®^ CT	MiniLab^®^ TLC test	HPLC content analysis adherence	Dissolution test compliance
	Isokin; Kinapharma Limited; Ghana	Ghana	May-14	400/80	Pass	Pass	No	Yes
	Deptrin 480; Dandams; Ghana	Ghana	Jul-13	400/80	Pass	Fail	No	No
	Co-Trimaxole; Letap Pharmaceuticals; Ghana	Ghana	Dec-12	400/80	Pass	Pass	No	No
	Co-Tri; Ayrton Drugs; Ghana	Ghana	Nov-13	400/80	Pass	Pass	No	Yes
	Sulfatrim Forte; Shalina Laboratories Pvt Ltd; India	Ghana	Aug-12	800/160	Pass	Pass	No	No
	Primpex^®^; SKG Pharmaceuticals; Nigeria	Nigeria	Mar-16	400/80	Pass	Pass	No	Yes
Co-trimoxazole tablets (*N* = 15)	Jutrim; Juhel Nigeria Ltd; Nigeria	Nigeria	Jan-14	400/80	Pass	Pass	No	No
	Jutrim; Juhel Nigeria Ltd; Nigeria	Nigeria	Feb-14	400/80	Pass	Pass	No	No
	Emtrim^®^; Emzor Pharmaceutical Industries Ltd; Nigeria	Nigeria	Jun-14	400/80	Pass	Pass	No	Yes
	Loxaprim; May and Baker Nigeria PLC; Nigeria	Nigeria	Aug-14	400/80	Pass	Fail	No	No
	Loxaprim; May and Baker Nigeria PLC; Nigeria	Nigeria	Oct-15	400/80	Pass	Pass	No	Yes
	Bactrim Forte^®^; Swiss Pharma Ltd; Nigeria	Nigeria	Oct-15	800/160	Pass	Pass	No	No
	Bactrim Forte^®^; Swiss Pharma Ltd; Nigeria	Nigeria	Oct-15	800/160	Pass	Pass	No	No
	Bactrim Forte^®^; Swiss Pharma Ltd; Nigeria	Nigeria	Aug-13	800/160	Pass	Pass	No	No
	Co-Trimoxazole 80 mg/400 mg Tablets; Glaxo Wellcome GmbH and Co; United Kingdom	United Kingdom	Jul-13	400/80	Pass	Pass	Yes	Yes
Total passed (%)					15/15 (100.0)	13/15 (86.7)		
Total failed (%)						2/15 (13.3)		
Total compliant with USP tolerance limits (%)							1/15 (6.67)	6/15 (40.0)
Total noncompliant with USP tolerance limits (%)							14/15 (93.3)	9/15 (60.0)

CT = colorimetric test; GSK = GlaxoSmithKline group of companies; HPLC = high-performance liquid chromatography; TLC = thin-layer chromatography; USP = U.S. Pharmacopeia.

The USP content limits state that 93–107% of the stated dose of components of co-trimoxazole must be measured for the tablets to be classified as compliant.

**Table 3:** Sensitivity and specificity of MiniLab^®^ tests for detecting drugs noncompliant with HPLC content analysis (frequency, proportion, and 95% exact confidence interval)

Antibiotic	Test	Sensitivity	Specificity
Amoxicillin	MiniLab^®^ CT	0/1	19/19
		0% [0.0–97.5]	100% [82.3–100.0]
	MiniLab^®^ TLC	0/1	17/19
		0% [0.0–97.5]	89.5% [66.9–98.7]
Co-trimoxazole	MiniLab^®^ CT	0/14	1/1
		0% [0.0–23.2]	100% [0.0–23.2]
	MiniLab^®^ TLC	2/14	1/1
		14% [1.8–42.8]	100% [2.5–100.0]

HPLC = high-performance liquid chromatography; MiniLab^®^ CT = colorimetric test; MiniLab^®^ TLC = semi-quantitative thin layer chromatography.
